# Effect of Paired Associative Stimulation on Corticomotor Excitability in Chronic Smokers

**DOI:** 10.3390/brainsci9030062

**Published:** 2019-03-15

**Authors:** Andrew P. Lavender, Hiroki Obata, Noritaka Kawashima, Kimitaka Nakazawa

**Affiliations:** 1 School of Physiotherapy and Exercise Science, Curtin University, Bentley WA 6102, Australia; 2 Department of Humanities and Social Sciences, Institute of Liberal Arts, Kyushu Institute of Technology, Fukuoka 804-8550, Japan; obata@dhs.kyutech.ac.jp; 3 Department of Rehabilitation of Movement Functions, Research Institute, National Rehabilitation Centre for Persons with Disabilities Tokorozawa, Saitama 359-0042, Japan; kws456123@gmail.com; 4 Department of Life Sciences, Graduate School of Arts and Sciences, The University of Tokyo, Komaba, Tokyo 113-8654, Japan; nak_kmtk@yahoo.co.jp

**Keywords:** transcranial magnetic stimulation, paired associative stimulation, smoking, cotinine

## Abstract

Chronic smoking has been shown to have deleterious effects on brain function and is an important risk factor for ischemic stroke. Reduced cortical excitability has been shown among chronic smokers compared with non-smokers to have a long-term effect and so far no study has assessed the effect of smoking on short-term motor learning. Paired associative stimulation (PAS) is a commonly used method for inducing changes in excitability of the motor cortex (M1) in a way that simulates short-term motor learning. This study employed PAS to investigate the effect of chronic cigarette smoking on plasticity of M1. Stimulator output required to elicit a motor-evoked potential (MEP) of approximately 1 mV was similar between the groups prior to PAS. MEP response to single pulse stimuli increased in the control group and remained above baseline level for at least 30 min after the intervention, but not in the smokers who showed no significant increase in MEP size. The silent period was similar between groups at all time points of the experiment. This study suggests that chronic smoking may have a negative effect on the response to PAS and infers that chronic smoking may have a deleterious effect on the adaptability of M1.

## 1. Introduction

The human sensorimotor cortex is described as being “plastic” which refers to its ability to adapt by reorganizing neural pathways in response to changes or injuries. Reorganization within the motor cortex can be stimulated by repetition of a simple task [1] and skill acquisition [2]. Excitability of the horizontal connections within the motor cortex can be increased, known as long-term potentiation (LTP) [3]. LTP refers to an enhancement of the amplitude of synaptic potentials, which can last for an extended period following repetitive activation of a specific neural circuit [4,5,6]. Conversely, long-term depression (LTD) describes the opposite effect, reducing the size of synaptic potentials [7]. Transcranial magnetic stimulation (TMS) activates intracortical fibres, which are oriented parallel to the scalp [8]. This leads to trans-synaptic activation of pyramidal output cells. Motor cortical pyramidal output cells receive somatosensory information at short latency via afferent fibres originating in subcortical and cortical regions [9]. These effects can be induced in the laboratory by pairing of peripheral electrical and TMS known as paired associative stimulation (PAS) [3]. The electrical stimulus for PAS, where a hand muscle is the target muscle, occurs at the median nerve near the wrist and the magnetic stimulator coil is positioned over the optimal point of the hand area of the motor cortex for that muscle known as the “hot spot.” The timing of the pulses relative to one another, known as spike-timing-dependent plasticity (STDP) is vital in that PAS protocols where a longer interstimulus interval (ISI) induces LTP and a shorter ISI results in LTD [4,10,11,12]. Cigarette smoking is known to affect *N*-methyl *D*-aspartate (NMDA) receptors by evoking dopamine release through activation of nicotinic acetylcholine receptors (nACHRs) in the prefrontal cortex [13]. A study by Jin et al. [14] found that the α-amino-3-hydroxy-5-methyl-4-isoxazolepropionic acid (AMPA)/NMDA ratio is increased in slices from ventral trigeminal area treated with nicotine suggesting that α7-nACHRs mediate this effect. Horizontal excitatory connections within the cerebral cortex are mainly glutamatergic [15,16] with larger fast AMPA and slower, low amplitude NMDA components. NMDA is a subclass of ionotropic receptors, central nervous system (CNS) receptors, which use glutamate as the primary excitatory neurotransmitter [17]. NMDA consists of a central ion channel and is of particular interest because it has several modulatory sites to which neurotransmitters and drugs can bind to affect the activity of the receptor. Studies have shown distinct distribution patterns in the adult rat brain compared with the developing brain which suggests different populations of neurons with unique NMDA receptor subunit compositions and distinct pharmacological properties [18,19]. NMDA receptors are recognised as being widely involved in neural and behavioural plasticity [20]. These include the development of tolerance, sensitization, and physical dependence to psychoactive drugs such as amphetamine, cocaine, barbiturates and nicotine. For a review, see Jain et al. [21].

Short-latency inhibition is enhanced, the silent period prolonged, intracortical facilitation reduced and motor-evoked potential (MEP) amplitudes lower in chronic smokers than non-smokers [22]. Neuroplasticity has been shown to be compromised in smokers during withdrawal and restituted by a nicotinic α4β2-receptor partial agonist [23]. So, the timing of any plasticity intervention such as transcranial direct current stimulation (tDCS) or PAS must be considered carefully when designing experimental interventions. Activation of NMDA receptors has been shown to be important in both LTP and LTD of cortical γ-aminobutyric acidergic (GABAergic) and cholinergic interneurons. STDP consists of presynaptic and postsynaptic elements and is now recognized as crucial for long-term synaptic plasticity [1,24,25]. It is therefore considered that perhaps the mechanisms by which PAS can enhance intracortical facilitation (ICF) and the size of MEP are somewhat reduced in smokers. A study into the effects of nicotine on neuroplasticity in healthy non-smokers showed that exposure to nicotine prolongs the enhancement of synapse-specific cortical excitability when induced by PAS25 and abolishes the PAS10-induced depression of cortical excitability [26]. This study also assessed transcranial direct current stimulation (tDCS) effects on cortical excitability reporting a trend towards reversal of tDCS-induced facilitation to inhibition under nicotine [26]. The authors propose that the focusing effect they reported is likely due to the different impact of cholinergic activation on the recurrent activation of afferent input to cortical neurons [26]. A similar investigation used nicotine spray rather than patches as it acts faster and is, therefore, more analogous to cigarette smoking [27]. In this study PAS and tDCS were, again, employed to induce focal and non-focal plasticity respectively. The authors found that nicotine spray abolished facilitatory plasticity following PAS25 and tDCS; however, PAS10 induced a diminution in excitability and tDCS-derived excitability reduction was delayed and comparatively weak [27]. Another study from the same group employed tDCS and PAS to assess the effect of nicotine withdrawal and exposure on neuroplasticity in smokers [28]. This study found that under nicotine withdrawal, tDCS and PAS25 failed to induce any facilitatory effect. However an inhibitory effect remained unaltered. The focusing effect shown in their previous study was not evident among the smokers in the follow up investigation [26,28]. More recently, Grundey et al. [29] assessed the effect of nicotine administration on calcium-dependent plasticity in non-smokers. The authors reported that nicotine administration abolished anodal tDCS-induced neuroplasticity and that under medium doses of a calcium channel blocker plasticity was re-established, suggesting the importance of calcium influx and calcium levels for nicotinic effects on LTP-like plasticity [29]. The present study applied PAS to assess the plastic characteristics of the motor cortex in smokers and compared these with non-smokers. It has been shown that ICF and cortical excitability are reduced in smokers [22] however, their response to PAS remains unclear. The present study compared the cortical plasticity of regular smokers with that of a non-smoker control group. Therefore, our hypothesis is that PAS will be less effective for generating cortical responses in the smoker group than in the non-smoker group.

## 2. Materials and Methods

### 2.1. Participants

Volunteers were recruited from among the staff and students of the local institution and the surrounding community. The procedure was approved by the Ethics Review Committee for Experimental Research with Human Subjects of the Graduate School of Arts and Sciences, The University of Tokyo, on 9 December 2011 (Project Identification Code: 224-2). This study was conducted in accordance with the statements of the declaration of Helsinki and all volunteers gave written informed consent before taking part in the study. Eleven non-smokers and 10 smokers, all self-reported right-handed males, aged between 27 and 43 years (Control = 34.9 ± 5.42, Smoker = 35.0 ± 4.81 (mean ± SD) volunteered for the study. Those describing themselves as chronic smokers also filled in a Fagerström smoking classification questionnaire to assess their tobacco addiction level. Fagerström dependence scores were classified as 0–2 = very low, 3–4 = low, 5 = medium, 6–7 = high and 8–10 = very high dependence [30]. All volunteers provided a urine sample for the assessment of cotinine, which is a marker used for the evaluation of recent and chronic consumption of cigarettes and nicotine. The experimental group consisted of 11 volunteers who were all long-term smokers and the control group was made up of 10 non-smokers. Experiments with smokers began no less than 90 min after smoking cessation but no more than 120 min since their last cigarette in order to avoid acute effects of smoking and onset of withdrawal symptoms [22].

Exclusion criteria included use of a cardiac pacemaker, metal implants in the head (excluding dental fillings), current use of any medication with contraindications for TMS such as antidepressants which alter cortical excitability, any current or previous neurological, psychiatric or internal disease, current or previous drug dependence (excluding nicotine for smokers), or alcohol abuse [22].

### 2.2. Transcranial Magnetic Stimulation

A Magstim 200 stimulator (Magstim, Whitland, Dyford, UK) and a figure-of-eight shaped coil with outer diameters of 9 cm were employed for all testing and PAS procedures. The coil was positioned over the left hand area of the motor cortex, tangentially to the scalp with the handle pointing backwards at an angle of 45° to the sagital plane. The coil was then adjusted slightly to find the optimal position for eliciting an MEP. The target muscle for this experiment was the first dorsal interosseous muscle (FDI) of the right hand. The intensity of stimulator output used for PAS and tests was assessed as the lowest percentage maximum stimulator output (%MSO) required to elicit an MEP of 1 mV peak-to-peak. This was done while subjects performed an isometric abduction of the index finger against a transducer at 10% maximum voluntary force (MVF). This stimulus intensity was then used for all pre- and post-tests as well as the PAS intervention. A block of 20 single TMS pulses was applied prior to PAS to establish baseline corticospinal excitability. This was applied at the predetermined %MSO so that the average MEP size across the 20 stimuli was approximately 1 mV. Blocks of 12 TMS pulses were applied at five-minute intervals for 30 min following PAS. The cortical silent period (CSP) was assessed from the same pre and post recordings and was measured from the point of the onset of the MEP to the point at which the EMG returned to the prestimulus mean [31]. The mean CSP for each time point was measured from the stimulus artifact to the point, after the MEP, where EMG amplitude returned to the prestimulus level. The prestimulus mean EMG amplitude was measured from 100 ms before the stimulus.

### 2.3. Paired Associative Stimulation

The PAS protocol used for the present investigation is a novel one. Subjects’ attention during the intervention has been shown to be an important factor for attaining optimal results [32]. Previous studies have employed various methods for ensuring subjects maintain concentration on the task throughout PAS. This usually involves counting the peripheral stimuli felt at the wrist or counting random electrical stimuli applied to a digit of the same hand as the target muscle [11]. This counting of peripheral stimuli has been commonly used for both PAS with the target muscle remaining relaxed and for PAS during which the subject is required to maintain a light (5% MVF) isometric contraction [33]. It is important that the isometric contraction be maintained without causing fatigue, and so it should be a very low contraction level and/or not allowed to continue for a long period of time. For the present investigation, subjects were required to perform an isometric abduction of the index finger using the target muscle, FDI, at 10% MVF. However, this was not maintained throughout the PAS procedure; rather, subjects abducted approximately 2 s before the paired electrical peripheral and magnetic cortical pulses and relaxed immediately after each paired pulse. By doing this the subjects were required to concentrate on the task to ensure they contracted at the sound of the buzzer and light on and relaxed after the PAS pulse at light off. PAS consisted of 180 TMS pulses at 0.2 Hz, each given 25 ms after an electrical pulse at 300% perceptual threshold, of the ulnar nerve near the wrist. This has the potential to optimize the effect of PAS by allowing a greater force production and requiring the subject to concentrate on the audible buzzer indicating the start of each contraction. An LED was employed to indicate, not only when to the start, but also when to stop the contraction and subjects also watched an oscilloscope to ensure the appropriate force was produced. Contractions began 2 s before each paired pulse and ended after the paired pulse, indicated by the LED being extinguished. This method allows for a lower %MSO to be used because of the higher force output. All subjects were given a few minutes before the start of the experiment to practice the task and all were able to perform it easily prior to beginning the experiment. Although the task was, therefore, relatively easy to perform it did require the subject to concentrate throughout the experiment.

Subjects were comfortably seated in an adjustable upright chair specially designed for TMS with an adjustable frame above the head for holding the coil. First the hand was placed in the manipulandum and secured in position using a Velcro strap. Subjects were then instructed to perform an MVF contraction of the first dorsal interosseous muscle by abducting the index finger as hard as they can against a custom built force plate for three seconds. Subjects were instructed to attempt to abduct the FDI at the distal joint without moving the hand and to maintain the force for three seconds. This MVF measure was then conducted again after one minute rest. The mean of the two measures was taken as the MVF. The hotspot for activation of the FDI was then located while the subject performed a continuous isometric abduction of the index finger at 10% MVF. After the hotspot was located, the coil and the head of the subject were locked into position in the frame. Because the subjects head and the coil were attached to the same frame, their position relative to each other could not change during the experiment. In addition to the frame holding the coil and head in the correct position, a three-dimensional position sensor (Polaris Vicra, NDI, Waterloo, Ontario, Canada) was used to track the coil position relative to the head of the subject to ensure the correct coil position was maintained throughout the experiment. Targets for the sensor were attached to a headband worn by the subject and the handle of the coil. These targets were detected using a three-dimensional camera placed about one meter in front of the subject at approximately 50 cm above the level of the head. Any change in the position of the head relative to the coil, were immediately detected by the position sensor which gave immediate feedback to the experimenter via a computer screen. If there were any changes the experimenter could instruct the subject so that he could return to the correct position. The subjects’ right arm was abducted slightly at the shoulder and rested on a table in front of them at slightly above waist level. The right hand of the subject was placed in a manipulandum designed and built in our laboratory for this and similar studies. A transducer was positioned against the distal interphelangeal joint of the right index finger. Electrodes were placed in a tendon-belly arrangement on the right first dorsal interosseous (FDI) and abductor digiti minimi (ADM) muscles. All EMG data was recorded using a Yokogawa W400 Measuring Station with the WE7000 software package (Yokogawa, Japan) and stored on a Dell XPS M1210 laptop computer for later offline analyses.

### 2.4. Statistical Analysis Procedures

Groups were compared for age, %MSO required to evoke motor evoked potentials (MEP) of 1mV and Cotinine scores using Students *t*-tests. Fagerström scores for the smokers were simply ranked and recorded. MEP amplitude data were normalised and changes in MEP amplitude following PAS and silent period were compared using a two factor mixed-design analysis of variance (ANOVA) with repeated measures for group (Control, Smoker) and time (pre, immediately post, then 5, 10, 15, 20, 25 and 30 min) with a significance level set at *p* < 0.05. Any differences between groups were assessed using Fishers PLSD post-hoc test. Data are expressed as mean ± standard error of the mean (SEM) unless otherwise indicated.

## 3. Results

### 3.1. Cigarette Consumption

**Figure 1:**
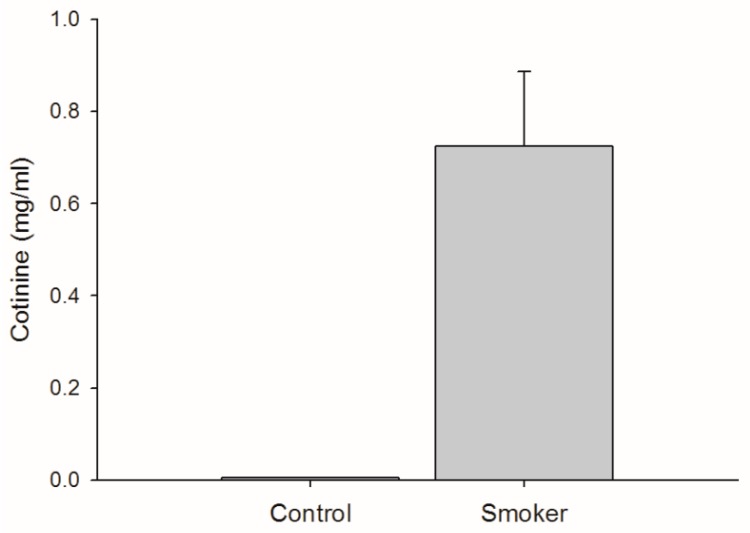
Cotinine content in urine samples from smokers and control subjects. A t-test comparison was done to compare groups and values found to show a statistically significant difference between groups.

Cotinine concentration was negligible for the non-smokers (< 0.005 ± 0.0 mg/mL) and typically higher for the smokers (0.746 ± 0.49 mg/mL) (*p* < 0.0001) (Figure 1). The mean ± SD of the Fagerström test scores was 2.82 ± 1.83 which classified the ten subjects’ addiction levels as very low (4 subjects), low (4 subjects) and medium (2 subjects). This level is similar to that of the subjects in a previous study of mildly addicted smokers [22].

### 3.2. Cortical Stimulation Parameters

The stimulator output level for tests and PAS was set at the %MSO which produced MEPs of approximately 1mV peak-to-peak. The %MSO for the groups were 47 ± 7.1% for smokers and 44 ± 7.8% for controls with a Students t-test showing no statistically significant difference between the groups (*p* = 0.20).

### 3.3. Force Output

It is important to assess the force output (10% MVF) of the subjects during both pre- and post-tests to ensure that the task was completed correctly in all experiments. Maximum voluntary force output varied between participants, so that the amount of force produced during the experimental procedure varied accordingly. There were no statistically significant differences between groups for force output across pre and post-tests (F1,7 = 1.30; *p* = 0.278). A one-way repeated measures ANOVA comparing pooled force output data from both groups showed no significant difference across time which indicates that force output was unchanged throughout the experiment for all subjects (F1,7 = 1.21; *p* = 0.302).

### 3.4. Motor Cortical Excitability

**Figure 2:**
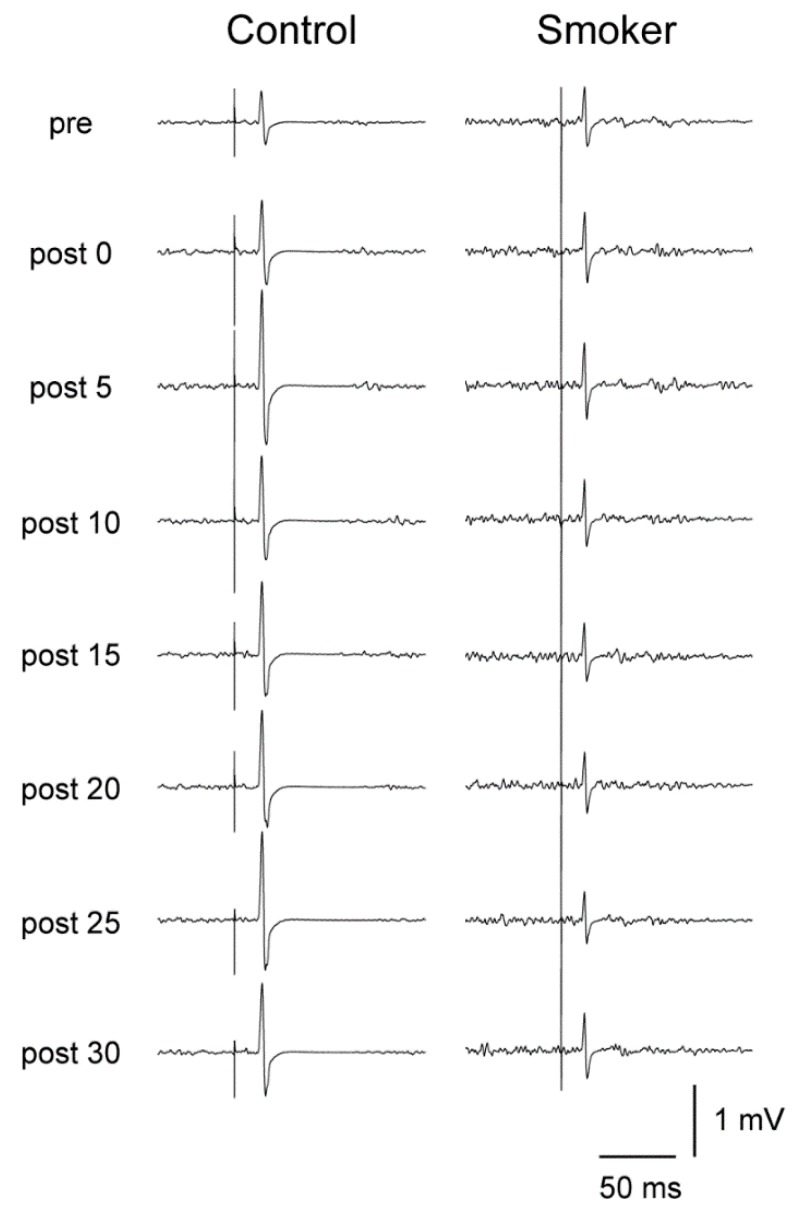
Comparison of effect of paired associative stimulation (PAS) on peak-to-peak motor-evoked potential (MEP) showing an example of raw data from one non-smoker control subject in the left-hand column and one smoker in the right-hand column.

The %MSO was set before beginning the experiment to elicit an MEP of approximately 1 mV. An example of raw data from one subject from each group which is typical of the overall group data is shown in Figure 2.

**Figure 3:**
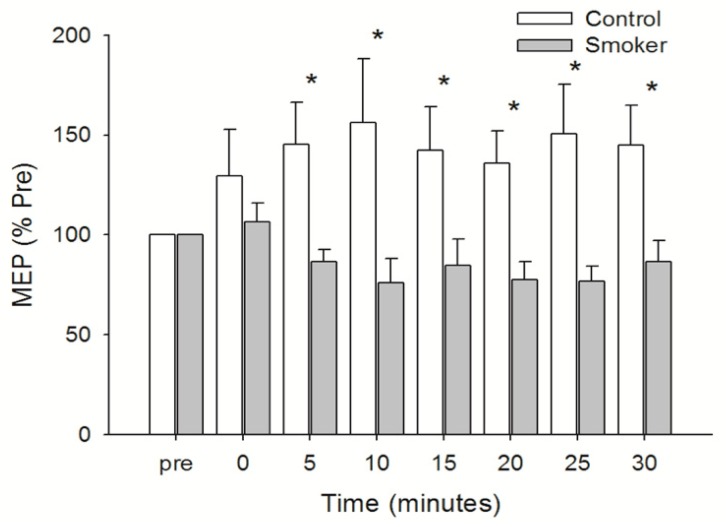
Peak-to-peak MEP for the Smoker and Control groups across time. This figure shows mean values ± standard error of the mean (SEM) of data normalized to the pre-exercise value. A two factor mixed-design analysis of variance (ANOVA) was performed and indicated a difference between groups. The differences were located using a Fischers PLSD post-hoc analysis and * indicates difference between groups at each time point after PAS.

Peak-to-peak MEP size was similar between the groups prior to PAS (Control = 0.84 ± 0.16 mV; Smoker = 0.95 ± 0.18 mV) (mean ± SD). A two factor mixed-design ANOVA comparing the groups over time showed a statistically significant interaction between group and time (F1,7 = 2.204; *p* = 0.038) and a Fisher’s PLSD post-hoc analysis showed the differences between the groups occurred at 5, 10, 15, 20, 25 and 30 min post-PAS. The control group increased by 30% immediately post-PAS and maintained a greater peak-to-peak MEP size throughout the 30 min post-PAS test period peaking at 10 min with MEPs 56% larger than the pretest (*p* = 0.044). Conversely, the peak-to-peak MEP size for the smoker group failed to increase substantially immediately after PAS (106% of pre-PAS) and remained less than the baseline level up to 30 min after PAS. The nadir was −24% which occurred 10 min (*p* = 0.614) after cessation of the PAS intervention (Figure 3).

### 3.5. Silent Period

**Figure 4:**
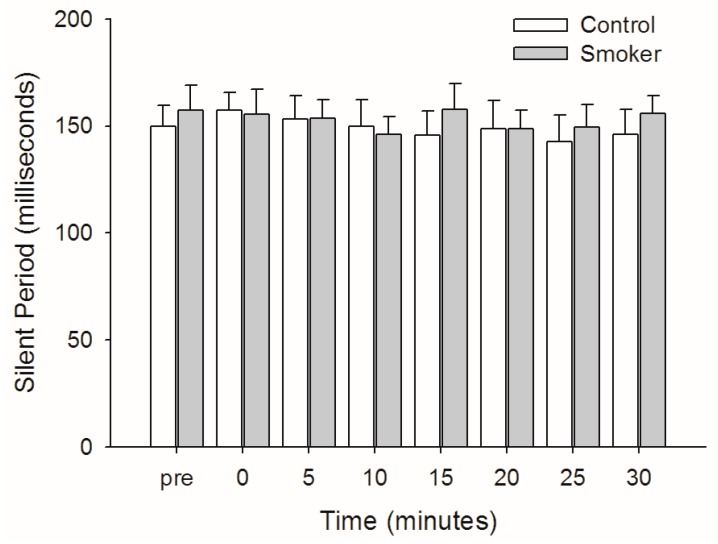
Mean silent period in milliseconds for control and smoker groups. Error bars indicate standard error of the mean.

As can be seen in Figure 4, the silent period was similar between the groups before PAS at 154 ± 28 ms for the non-smokers and 163 ± 33 ms (mean ± SD) for the smokers. This remained unchanged following PAS for both groups (F1,7 = 0.614; *p* = 0.743).

## 4. Discussion

The present study found that the stimulus intensity required to elicit an MEP of 1 mV was similar between the groups, and in line with the study by Lang et al. [22]. 

The level of magnetic stimulator output (%MSO) required for eliciting a 1 mV MEP response, although slightly higher for the smokers, was statistically similar between the groups. This demonstrates comparable baseline excitability between the experimental and control groups while the muscle is performing a light isometric contraction, although previous research has found a reduction in cortical excitability at rest in smokers compared with non-smokers [22]. It was thought that the smokers would respond in a positive manner to the PAS, and perhaps the control group would have a response indicating efficient adaptable connections along the corticospinal pathway for both groups. This was not the case among the smokers as their MEP amplitude immediately following PAS increased by only 6%. As a group, the level of excitability as indicated by MEP size for the smokers decreased slightly within five minutes and remained low up to 25 min post-PAS following the slight increase immediately after PAS. This decrease was not large enough to register a long-term depression (LTD) effect in the level of cortical excitability compared with the baseline measure; however, the slight reduction persisted for several minutes after PAS. A significant reduction in the size of MEP following PAS has been shown previously using PAS with an interstimulus interval (ISI) of 10 ms [24]. It is difficult to reconcile this slight but not statistically significant decrease in cortical excitability in the smokers. Long-term synaptic plasticity is known to be affected by PAS in either a positive, LTP, or negative, LTD, manner governed by STDP [24]. STDP requires that in order to evoke LTP like changes in cortical excitability for the FDI, the ISI between the afferent electrical and cortical magnetic stimuli should be 25 ms and for LTD effects the ISI should be 10 ms [34]. Therefore, since the timing of the pulses was 25 ms for both groups a positive change in cortical excitability was expected. The results of the smokers in this study may be explained by differences in cerebral blood flow dynamics in smokers compared with non-smokers. Chronic smoking is known to lead to impaired endothelial function due to a decrease in the formation of nitric oxide and an increase in the degradation of nitric oxide through the generation of oxygen free radicals. Nitric oxide from efferent nitrergic nerves is also involved in vasodilation, increased regional blood flow, and hypotension that are impaired through nitric oxide sequestering by smoking-induced factors [35]. It has previously been shown that regional cerebral blood flow (rCBF) increases significantly during movement in a goal directed task. Winstein et al. [36] showed clear increases in rCBF in the left sensorimotor, dorsal and ventral premotor, mesial frontal and parietal cortex during a target-aiming task in which the subjects moved a stylus gripped like a pen in the right hand. Sidtis et al. [37] showed that resting rCBF is significantly affected by the task with which it is associated during an experimental imaging session. Another, more recent, investigation found that motor skill training significantly increases rCBF both during task performance and at rest [38]. Therefore, if rCBF is reduced in smokers perhaps the learning effect induced by PAS in our study may be rendered less effective. Another possible factor contributing to the difference between the groups in this study is that although the initial %MSO required to produce an MEP of 1 mV was similar between the groups, the stimulation level, or %MSO, required to elicit a positive paired associative stimulation (PAS) response in the smokers may be greater than for non-smokers.

Impaired GABA-mediated mechanisms have been observed in clinical contexts, for example coeliac disease, providing further evidence that a disinhibited M1 is involved even in the absence of a clear motor impairment [39,40]. Interestingly, an impaired motor plasticity induced by inhibitory low-frequency rTMS has also been shown in patients with restless leg syndrome (RLS) [41,42]. RLS has been described by Lanza et al. [41] as “a sensory-motor network disorder with a modified excitability of a complex corticospinal drive involved in both somatosensory perception and movement generation.” Perhaps these disorders cause a similar impairment to GABAergic mediated activation as chronic smoking resulting in an impaired response to PAS in the smoker group compared with controls. Lang et al. [22] have shown that chronic smokers display enhanced short-latency afferent inhibition (SAI), prolonged inhibitory silent periods, reduced intracortical facilitation and lower active MEP amplitudes compared with non-smoker controls. The authors suggest that this is an indication of differences in excitability between the groups at the cortical level. Perhaps, although the pre-test %MSO required for eliciting MEPs of approximately 1 mV was similar between the groups, smokers may require a greater level of stimulator output in order to achieve the same changes in MEP size with PAS. Interestingly, the silent period for the smokers in the present investigation was slightly longer but not statistically different from that of the control subjects. However, the Fagerström scores, which rate our subjects as mildly addicted, suggest that the addiction level of our smokers was similar to those in the study by Lang et al. [22]. The cortical silent period (CSP) is a complicated measure and can be affected by factors including stimulus intensity [43] and modulated by short interval intracortical inhibition (SICI) and ICF paired pulse paradigms [44]. The CSP following an MEP is mediated by GABA-B receptors and in concert with SICI circuitry which is also GABAergic, although GABA-A mediated, plays an important role in motor performance [45]. Since smoking likely enhances somatosensory inhibition of the motor cortex by activation of cholinergic synapses, as suggested by Lang et al. [22], it is possible that the PAS had an excitatory effect on the inhibitory circuits of the smokers resulting in reduced MEPs following PAS.

Another important consideration regarding changes in cortical function is the effect of cognitive functioning on TMS assessments. In recent years, the use of TMS techniques to assist in diagnosis of pathologies, such as dementia, and efforts to improve our understanding of the neurophysiological mechanisms associated with normal ageing and neurodegenerative disorders provides a challenging direction for researchers [46]. For example, a longitudinal study by Pennisi et al. [47] addressed the changes in M1 excitability in patients with vascular cognitive impairment absent of dementia, vascular depression patients and controls. The authors reported that ICF was enhanced in the patients with vascular cognitive impairment absent of dementia compared with the other groups but became similar to the other groups at follow up two years later. Enhanced glutamate-related plasticity was suggested as the reason for the high level of ICF at baseline that provided some protective effect against cognitive decline in this group [47]. Synaptic plasticity of M1 is likely facilitated by activation of NMDA glutamate receptors, which has been shown previously by an increase in ICF in patients with cardiovascular risk factors and chronic subcortical ischemic disease. Bella et al. [48] showed a significant decline in rMT in patients with vascular cognitive impairment absent of dementia with a trend towards a decrease in facilitation with a conditioning stimulation at longer interstimulus intervals. It is suggested that inputs from glutamatergic pathways are not functioning in the patients. For a recent review on the use of TMS as a diagnostic tool for providing insights into the pathophysiology of neurological and psychiatric diseases through the assessment of corticospinal excitability, please see the article by Lanza et al. [49].

### Limitations

This study does have some limitations. In this study we used a novel approach to PAS. We considered previous protocols and tried this method as it requires the subject to activate the muscle at a higher intensity than other active protocols, while avoiding fatigue, and gave them a task that kept them focused on the intervention in an active manner. While this approach does not appear to be any less effective than previously used methods, it does not seem to be any more effective either. Including rCBF or magnetic resonance imaging (MRI) measures of cortical function may have provided further useful data for this investigation and aided in providing a more comprehensive assessment of the effects of chronic cigarette smoking on cortical function. This study was limited to the motor cortex and provides some insight to these effects. In the present study volunteers were not patients undergoing any treatment for neurological disorders and were not screened for cognitive deficits, nor were they assessed by MRI in order to exclude neurophysiological deficits or radiological lesions. For future studies, researchers should consider including such screening if the equipment is available since neurological disorders may affect results [41,42,43]. Including a standardized test of handedness, such as the Edinburgh Handedness Inventory [50], rather than simply asking the subjects which hand they considered as dominant would be appropriate for a study such as this. Further directions for research may employ measures of bilateral TMS recording to investigate the potential for intercollosal activity to affect plasticity in order to add further to the body of knowledge on this important topic. According to the guidelines provided by Rossini et al. [31], the most precise method for estimation of MEP size is by taking a ration between the maximal MEP and the maximal, distally evoked compound motor action potential (CMAP). This approach is thought to reflect central mechanisms that contribute to the amplitude of an MEP, thereby minimizing inter-subject variability seen with the MEP alone and improve the accuracy of data.

## 5. Conclusions

This study was carried out to ascertain whether there is a difference in the extent to which the motor cortex responds to paired associative stimulation between healthy chronic smokers and healthy non-smokers of similar age. The non-smokers responded well to PAS and the smokers did not. Indeed the smokers failed to respond at all to the PAS intervention. We have suggested some possible reasons for this result but clearly further research into the effects of chronic cigarette smoking on cortical excitability and the effectiveness of PAS is warranted.
